# Length of hospital stay and factors associated with very-low-birth-weight preterm neonates surviving to discharge a cross-sectional study, 2022

**DOI:** 10.1186/s12887-024-04532-5

**Published:** 2024-01-26

**Authors:** Yimenu Mehretie, Ashenafi Tazebew Amare, Geta Bayu Getnet, Birhanu abie Mekonnen

**Affiliations:** Department of Pediatrics and Child Health, School of Medicine, College of Medicine and Health Science, University of Gondar, Po. Box 196, Gondar, Ethiopia

**Keywords:** Hospital stay, Very low birth weight, Preterm neonates, Surviving, Discharge

## Abstract

**Background:**

The length of hospital stay of very-low-birth-weight neonates (birth weight < 1500 g) depends on multiple factors. Numerous factors have been reported to influence the length of hospital stay (LOS). The objective of this study was to identify the length of hospital stay and associated factors among very-low-birth-weight preterm neonates.

**Method:**

A hospital-based, cross-sectional study was conducted. Data was collected using a pretested, structured questionnaire from April 1 to November 30, 2022. The data was entered using Epidata and Stata version 15.1. The frequencies, mean, median, and interquartile range were used to describe the study population about relevant variables. A linear regression model was used to see the effect of independent variables on dependent variables.

**Result:**

About 110 very low-birth-weight preterm neonates who survived to discharge were included in the study. The median birth weight was 1370 g, with an IQR of 1250–1430. The mean gestational age was 32.30 ± 1.79 weeks. The median length of hospital stay was 24 days, with an IQR of 13.5–40. The gestational age, type of initial management given, and presence of complications had a significant association with the length of hospital stay for VLBW preterm neonates.

**Conclusion:**

The median hospital stay was 24 days. The gestational age, presence of complications, and type of initial management given were associated with LOS for VLBW preterm neonates. The length of the hospital stay of the VLBW preterm neonates can be reduced by applying the standards of care of very-low-birth-weight preterm neonates.

## Background

Low-birth-weight neonates are those who weigh less than 2500 g, whereas a very-low-birth weight is one who weighs less than 1500 g [1]. Around 17.3% of neonates in Ethiopia are born underweight. The factors that have been linked to very-low-birth-weight include maternal age < 20 years, maternal age > 34 years, pre-eclampsia, gestational DM, preterm, and gestational age < 37 weeks [2].

The length of stay (LOS) of very low birth weights in the hospital was influenced by their gestational age (GA) and any associated medical conditions, including bronchopulmonary dysplasia (BPD), repeated apneic episodes, or tube feeding [3]. There were relatively few evidence-based, consensus-based criteria for when to discharge a very preterm infant, despite the fact that many therapies in this high-risk group adhered to standardized recommendations in this regard. As a result, the discharge time would be varied. Therefore, a VLBW infant might be discharged home at various times in different settings, even though they have the same GA and weight [4].

A prolonged stay at the hospital lengthened exposure to risks associated with the hospital environment, including nosocomial infections [5, 6]. Studies also showed that the length of hospital stay of preterm neonates increased the hazard of death [7]. On the other hand, when a very low-birth-weight preterm neonate stayed less than the median length of hospital stay, there would be an associated increase in mortality [8]. In addition, the prolonged stay of the neonate in the neonatal intensive care unit (NICU) interfered with the development of relationships between parents and their children [9]. From a health system perspective, longer LOS reduced availability of beds and increased the health care cost [10–12].

The very-low-birth-weight preterm neonates had delicate, immature organs and required their own intensive care unit. Deciding the timing of discharge was challenging since there was no set protocol. There was a wide variation in discharge recommendations throughout the world. It was the same in the setup where this research held. The preterm neonates of the same birth weight had not discharged on the same day, which suggested the presence of factors contributing to their respective hospital stays.

Prolonged hospital stay had a multifaceted effect on the neonates with very low-birth-weight. It was necessary to identify the contributing factors to the additional days. The expected median age of hospital stay for very low-birth-weight preterm neonates was not previously determined in the setup where this research was done. In addition, knowing the contributing factors would be one of the solutions to the problem. The aim of this research was to determine the length of hospital stay and factors that were associated with length of hospital stays in very low-birth-weight preterm neonates surviving to discharge.

## Method

### Study design and setting

A hospital-based cross-sectional study design was conducted at the University of Gondar comprehensive specialized hospital (UoGCSH) neonatal intensive care unit. UoGCSH is a referral, teaching, and research hospital in North Gondar, Ethiopia. The critical care unit, oncology unit, neonatology unit, cardiac unit, well-child care service, and outpatient department are among the pediatrics and child health department. The neonatal intensive care unit is adjacent to the labor ward and accepts referrals from various healthcare facilities in the UoGCSH catchment area. The NICU ward has three distinct spaces: the term, preterm, and kangaroo mother care (KMC) rooms. The discharge criteria of very-low-birth-weight preterm neonates are weigh ≥ 1500 g, normal temperature, fed on breast milk on their own, and didn’t experience complications.

### Inclusion and exclusion criteria

The study included all preterm neonates with very low birth weights who were discharged alive. Preterm neonates excluded were referral neonates from other facilities after the seventh day of postnatal age and transferred out of the hospital before meeting the discharge criteria.

### Sample size determination and sampling procedure

The final sample size was 110, with a population percentage of 7.82%, a confidence interval of 95%, and a margin of error of 5%. Due to a lack of data, the research included all VLBW preterm neonates who were discharged alive during the study period.

### Operational definition

*Neonate* is an infant younger than 28 days of age.

*Preterm* is neonate born before 37 completed weeks from the first day of their last menstrual period.

*Early/very preterm* is preterm neonate born with 28-31 weeks of gestation from the first day of their last menstrual period.

*Moderate preterm* is preterm neonate with 32–33 weeks of gestation the first day of their last menstrual period.

*Late preterm* is preterm neonate with 34–36 weeks of gestation the first day of their last menstrual period.

*Very low-birth-weight* is neonate born with birth weight of < 1500 g.

*Length of stay* is length of time between the neonate’s admission and survived discharge from the unit to home.

*Maintenance fluid* is fluid that is given via intravenous line that contains fluid, glucose and electrolyte.

*Mixed feeding* is neonate who was taking both expressed breast milk and formula at any time during his/her hospital stay.

### Data collection tools and procedure

We employed a standardized, tested-in-advance English questionnaire. Under the guidance of the investigators, two medical interns were chosen to gather the data. Secondary data (maternal and neonate records) and in-person interviews with the mothers at the time of discharge were the sources of information. The mothers were directly interviewed face-to-face, and the mothers’ basic demographic information was collected. Weight, gestational age, complications, length of stay, post-menstrual age of the neonate, and mother sociodemographic variables were prioritized during data collection.

### Data quality control

Prior to the actual study period, a pretest using 5% of the sample size was carried out to evaluate the validity and accuracy of the questionnaire. The data collectors were trained on the purpose of the study, its applicability, information confidentiality, respondents’ rights to informed consent, and how to complete the questionnaire. To verify the accuracy and consistency of the data collected, the primary investigator and the supervisor often reviewed the data gathering procedure.

### Data processing and analysis

With dummy tables, the questionnaire was given the initial code. The data was then input using the statistical program Epidata version 4.6 and analyzed with Stata version 15.1. Data cleaning, accuracy and consistency, and missing values and variables were checked. The frequency and percentage summaries of categorical variables were used. The mean and median were used to characterize continuous variables. After performing all assumptions of linear regression, we undertook multiple linear regression to determine factors associated with length of hospital stay. The outcome variable was transformed with a square root to make it non-skewed. A *P*-value of < 0.05 and a 95% confidence interval were used to identify the statistically significant variables.

## Result

### The socio-demographic characteristics

There were a total of 175 very low-birth-weight preterm neonates admitted over the study period. From which, 110 were discharged alive with a 63% survival rate. The male-to-female ratio of the study participants was 1.5:1. The median family income was 8,250 Ethiopian birr (ETB), with an interquartile range (IQR) of 5,000–12,250 ETB. The minimum and maximum family income were 1100 and 25,000 ETB, respectively, with 50% less than the median. Mothers who came from rural areas were 82 (74.55%); of these, 20.73% had no formal education. The mean gestational age was 32.3 ± 1.79 weeks. The minimum and maximum gestational ages were 29 and 36 weeks, respectively. The median birth weight was 1,370 g, with an IQR of 1,250.0–1,430.0. The maximum and minimum birth weights were 1000 g and 1,495 g, respectively (Table 1).

**Table 1:** Sociodemographic characteristic of VLBW preterm neonates and their mothers admitted to UOGCSH, 2022

Variable	Category	Frequency	Percentage
Gestational age	Early preterm	58	52.73
	Moderate preterm	22	20
	Late preterm	30	27.27
Birth weight	< 1370gm	54	49.1
	≥ 1370gm	56	50.9
Corrected GA	< 36 weeks	67	60.9
	≥ 36 weeks	43	39.1
Sex	Male	66	60
	Female	44	40
Maternal age	< 20	6	5.45
	20–35	57	51.82
	> 35	47	42.73
Address	Urban	28	25.45
	Rural	82	74.55
Level of education	No formal education	19	17.27
	Primary school	56	50.91
	Secondary school	29	26.36
	College and above	6	5.45

### Clinical characteristics

Among mothers from rural areas, 19.5% gave birth at home. However, all urban mothers gave birth at health institutions. The anthropometric measurements of neonates revealed that 89 (80.91%) were appropriate for gestational age (AGA), and the remaining 21 neonates (19.09%) were small for gestational age (SGA). There was no neonate large for gestational age (LGA). Ninety-five percent of the SGA preterm neonates were late-preterm.

**Fig. 1:**
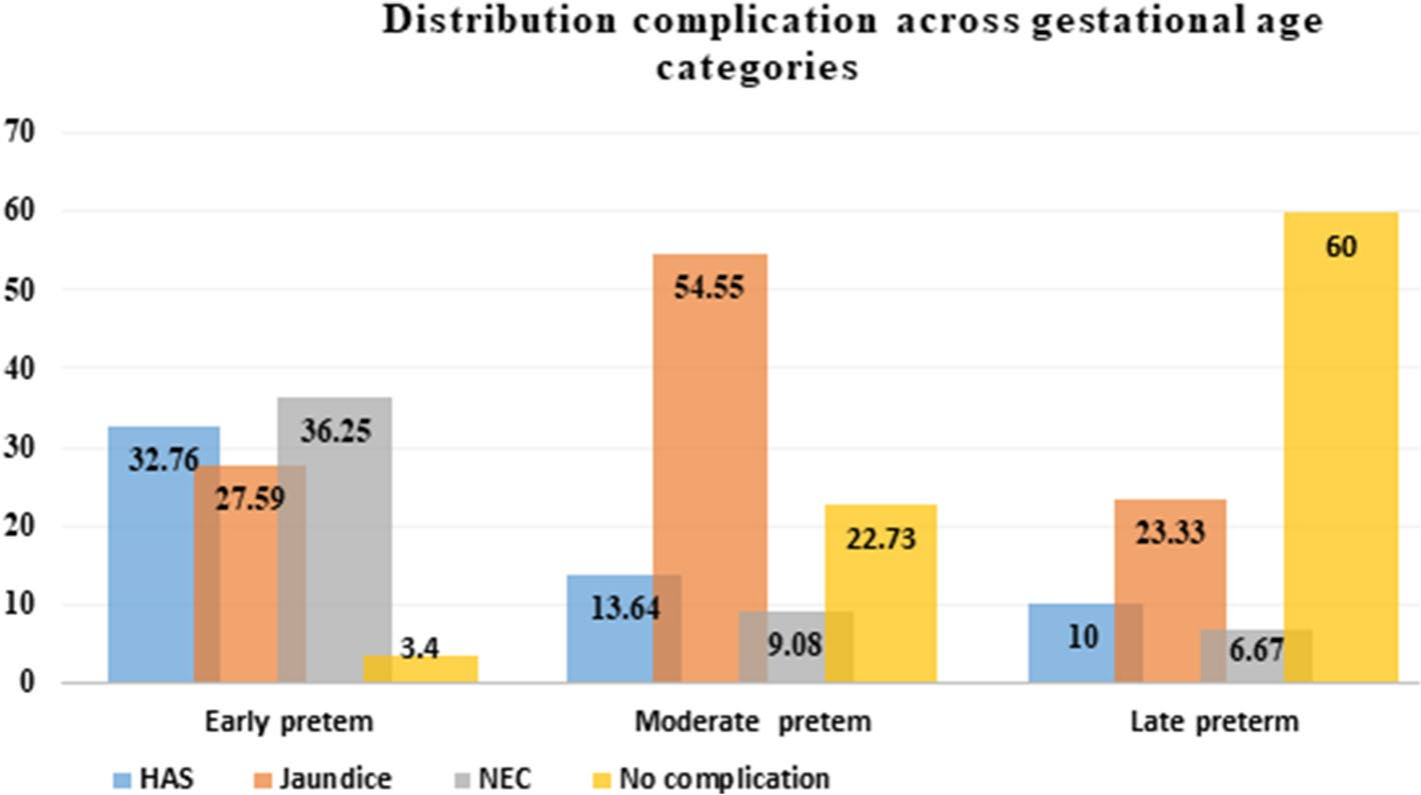
Distribution of complications across gestational age categories

The three most common admission diagnoses were respiratory distress syndrome (56.3%) and early-onset neonatal sepsis (31.8%), followed by very low birth weight (4.5%). The very low-birth-weight preterm neonates developed different complications at the hospital (Table 2 and Fig. 1). The median postnatal age at the initiation of feeding (expressed breast milk) was 43.5 h, with an IQR of 24–72 h. However, seven neonates were started on expressed breast milk (EBM) feeding at the first hour(h) of postnatal age, and just one neonate began EBM at 240 h (10 days) of age.

**Table 2:** Clinical characteristics of VLBW preterm neonates and their mothers admitted to UOGCSH, 2022

Variables	Category	Frequency	Percentage
Parity	Primiparous	32	29.09
	Multiparous	69	62.73
	Grand multiparous	9	8.18
Place of delivery	Inborn	78	70.91
	Referral	20	18.18
	Home	12	10.91
Mode of delivery	SVD	49	44.55
	Assisted	11	10.00
	C/S	50	45.45
ANC follow up	Yes	94	85.45
	No	16	14.55
HIV status	Positive	5	4.55
	Negative	105	95.45
Age at the start of EBM	< 49 h	75	68.18
	≥ 49 h	35	32.82
Feeding type	Expressed breast milk	86	78.18
	Mixed	24	21.82
Type of Management	CPAP	68	61.82
	Intravenous antibiotics	37	33.64
	MF fluid only	5	4.55
Complications	Hospital acquired sepsis	25	22.72
	Necrotizing enterocolitis	25	22.72
	Jaundice	35	31.82
	No complications	25	22.72

### Length of hospital stay

The median length of hospital stay of VLBW preterm neonates was 24 days, with an IQR of 13.25–40 days and a range of 2–78 days. 48.18% of the study participants had hospital stays longer than the median. The mean corrected gestational age (post-menstrual age (PMA)) at discharge was approximately 36.2 ± 0.18 weeks (95% CI, 35.82–36.52) but varied from 33 to 41 weeks. Six (5.5%) of the study subjects were discharged at 33 weeks postnatal age, while the rest discharged after 34 weeks. Among participant neonates, 32.7% discharged at a PMA of 35 weeks. Around 43 (39.1%) of VLBW preterm neonates discharged at ≥ 37 weeks of post-menstrual age.

**Fig. 2:**
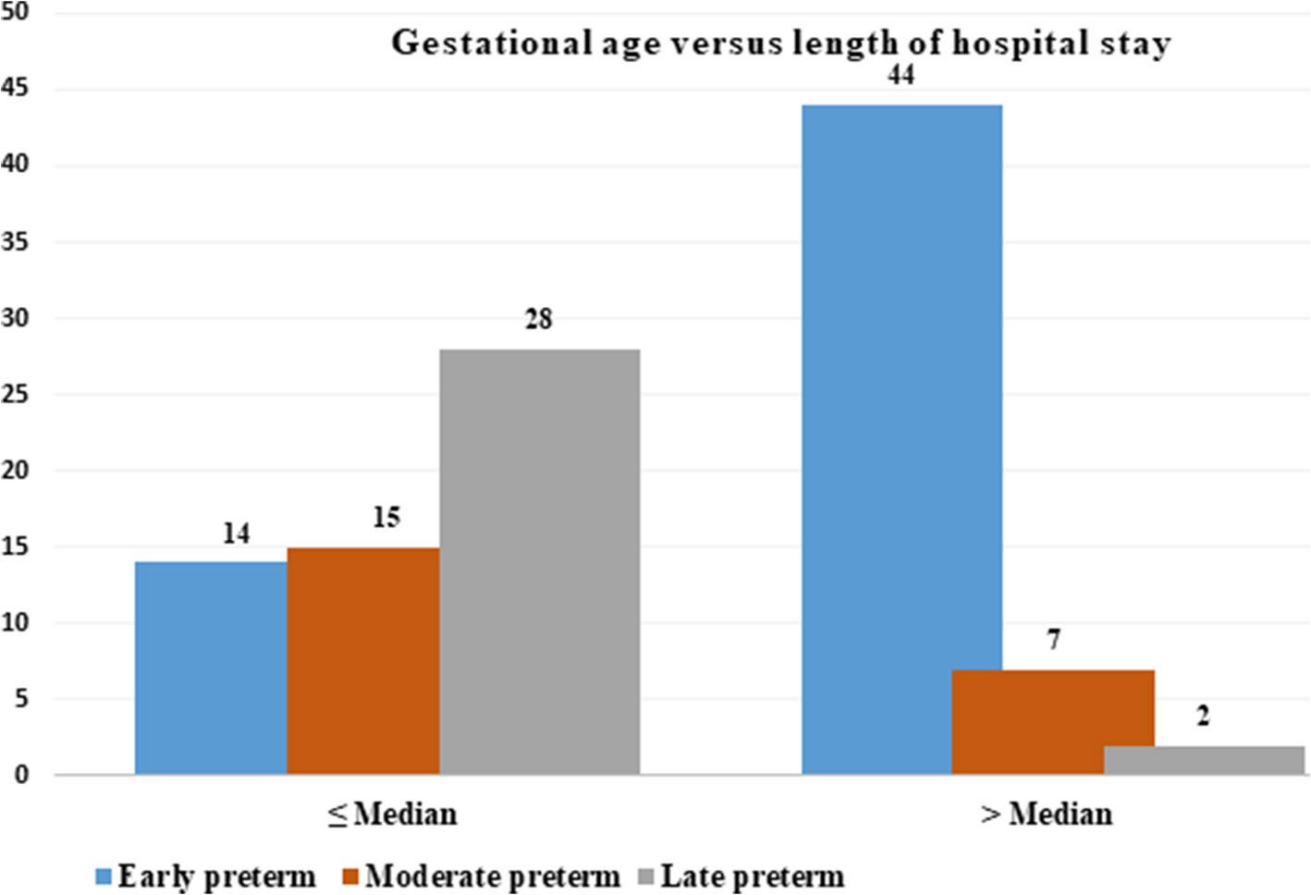
Gestational versus length of hospital stay

Among the study participants discharged before 24 days (median), 22.72% had no complication, while 29% had complications. All study subjects (48.18%) who stayed more than 24 days (median) had either of the complications (Fig. 2).

### Factors associated with length of hospital stay

From the summary of the multiple linear regression model analysis, length of hospital stay was explained by the independent variables by about 94%. We checked each independent variable against the dependent variable, and complications, gestational age, time to reach maximum feeding, age at feeding initiation, management type, birth weight and address were associated with the dependent variable. Multiple linear regression was done to identify the associated factors of length of hospital stay. Accordingly, gestational age, type of management given, and presence of complications were significantly associated with length of hospital stay for very low-birth-weight preterm neonates (Table 3).

**Table 3:** Multiple linear regression analysis of factors associated with length of hospital stay for very low birth weight preterm neonates admitted to UoGCSH, 2022

Variable	Category	COR	AOR	*P*-value
Address	Urban	-	-	-
	rural	0.52(0.23–1.15)	1.17(0.97- 1.42)	0.098*
Gestational age (in weeks)		0.44(0.39–0.496)	0.554(0.52—0.595)	0.000***
Management type	CPAP	57.2(16.08–203.23)	1.79(1.11- 2.87)	0.017**
	Antibiotics	5.824(1.58–21.46)	1.61(1.07–2.42)	0.024**
	Maintenance fluid	-	-	-
Age at feeding initiation( in hours)		1.03(1.02–1.04)	1.002(0.999—1.01)	0.26
Time to reach maximum feeding (in hours)		1.28(1.24–1.33)	1.01(0.98—1.04)	0.463
complication	Hospital acquired sepsis	46.94(24.17–91.14)	1.566(1.12- 2.19)	0.009***
	Jaundice	5.27(2.85–9.45)	1.41(1.11- 1.80)	0.006***
	Necrotizing enterocolitis	27.88(14.36–54.15)	1.647(1.18- 2.296)	0.004***
	No complication	-	-	-

*-90% CI

**-95% CI

***-99% CI

There was a 45% decrease in the length of hospital stay for every one-week gestational age in VLBW preterm neonates (AOR = 0.55, 95% CI (0.52–0.595)). The length of hospital stay of VLBW preterm neonates managed with continuous positive airway pressure (CPAP) was increased by a factor of 1.8 (AOR = 1.79, 95% CI: 1.11–1.87) and antibiotics by 1.6 (AOR = 1.61, 95% CI: 1.07–2.42) as compared with those managed with maintenance fluid only. The length of hospital stay was increased by the odds of 1.57 for hospital-acquired sepsis (HAS) (AOR = 1.57, 95% CI: 1.22–2.19), 1.65 for necrotizing enterocolitis (NEC) (AOR = 1.65, 95% CI: 1.18–2.296), and 1.41 for jaundice (AOR = 1.41, 95% CI: 1.11–1.80) as compared to VLBW preterm neonates with no complications.

## Discussion

The findings of this study showed that the median length of hospital stay was 24 days. The hospital length of stay (LOS) of VLBW preterm neonates was significantly associated with the gestational age, type of management given, and the presence of complications.

The overall median LOS of VLBW preterm neonates was found to be 24 days, with an IQR of 13.25–40 days. This study was supported by a study conducted at Johannesburg’s Chris Hani Baragwanath Academic Hospital, which found that the median hospital stays of VLBW neonates were 39 days [3]. In addition, research done in King Fahad Medical City tertiary hospitals showed that the median length of stay was 14.5 days, which was in line with our study [13]. The other research that supported this result was done at the Loma Linda University (LLU) Children’s Hospital, where the average length of hospital stay was 24.01 days [14].

However, the LOS for VLBW preterm newborns who survived to discharge was found to be 62 days in the research conducted at the National Cheng Kung University Hospital in Taiwan. In addition, research done in European countries on the duration and time trends in hospital stay for very preterm infants across European regions showed the mean length of hospital stay was between 54 and 70 days [15]. Both the Taiwanese and the European countries studies did not support the findings of this study. The difference in the hospital length of stay in Taiwan and Europe with this study was due to the study participants, in which very preterm and very-low-birth weight (weight < 1000 gm) preterm neonates were included in the Taiwan and European studies. So the length of hospital stays in Taiwanese and European countries was longer than the current study.

Research done on the morbidity and mortality patterns of preterm low birthweight neonates admitted to referral hospitals in the Amhara region and in the other five Ethiopian hospitals showed the mean length of hospital stay was 9.82 and 7 days, which was lower than this study [8, 16]. This was because the study participant included in the study were low birth weight preterm neonates (birth weight < 2500gm) and the sample size was also larger as compared to the current study. These differences were due to the study participants included low-birth weight preterm neonates (birth weight < 2500 g), and the sample size was larger as compared to the current study.

Factors associated with the length of hospital stay of very low-birth-weight preterm neonates were gestational age, management type, and complications. For every one week, regardless of the gestational age of the VLBW preterm neonates, the length of hospital stay decreased by 45%. The odds of prolonged hospital stay for preterm neonates were 2.8 compared to their term counterparts in research done at Kenyatta National Hospital [4]. According to South African research, for each one-week increase in gestational age, the length of hospital stay of VLBW preterm neonates decreased by 0.4 days [3].

The other factor associated with the length of hospital stay was the type of management given to the VLBW preterm neonates. Neonates managed with continuous positive airway pressure (CPAP) and antibiotics were highly likely to stay longer than neonates only given supportive care (maintenance fluid). Research done in Ankara, Turkey, showed very low-birth-weight neonates who were on ventilation (both invasive and non-invasive) increased their length of stay as compared to those who did not get ventilation (respiratory treatment) [17]. The rate of discharge of preterm neonates decreased by 45.7% with the development of RDS in research done on predictors of the length of stay for preterm infants in Ethiopia [16].

Antibiotic intake was also another associated factor for the length of hospital stay for very low-birth-weight preterm neonates as compared to those receiving supportive care only. This might be due to antibiotics given to VLBW preterm neonates that had nosocomial infection and necrotizing enterocolitis (NEC), which were identified as associated factors of the length of hospital stay in this study.

Complication was one of the important associated factor for VLBW preterm neonates as compared to neonates without complication. Hospital acquired sepsis increased the length hospital of stay by Complication was one of the associated factors for VLBW preterm neonates as compared to neonates without complications. Hospital-acquired sepsis (HAS) increased the length of hospital stay by odds of 1.6 compared to those without complications. This result was supported by research conducted in Johannesburg’s Chris Hani Baragwanath Academic Hospital, which showed health care-associated infections increased the length of hospital stay by odds of 31.9. The other research that supported the current study was done in AL Zahra Hospital, Tabriz, Iran. It showed that bacterial colonization and nosocomial infections were factors associated with prolonging hospital stays for very low-birth-weight preterm infants [18]. Necrotizing enterocolitis and nosocomial infection contributed to prolonged hospital stays for very low-birth-weight preterm neonates [19, 20]. The main reason that complications were an associated factor in hospital stays for VLBW neonates was that most complications happened at some point of admission and after the initiation of the initial management, and the new management for the complications would take an additional period of time.

### Limitation of the study

The study has limitations in analyzing clinical conditions like hypothermia, glucose level, anemia, and risk factors for preterm delivery like PROM, gestational DM and gestational hypertension, and maternal anthropometry which might have association with LOS.

## Conclusion

The median hospital stay for VLBW was 24 days. The length of hospital stay was inversely related to the gestational age of neonates. The gestational age, presence of complications, and type of management given were associated with the length of hospital stay for VLBW preterm neonates.

### Recommendation

The length of the hospital stay of the VLBW preterm neonates can be reduced by applying the standards of care for very-low-birth-weight preterm neonates. Further research can be done in this area to identify other determinants of the length of stay.

## Data Availability

No datasets were generated or analysed during the current study.

## Abbreviations

AGA: Appropriate for gestational age
ANC: Antenatal care
BMI: Body mass index
ETB: Ethiopian birr
BPD: Bronchopulmonary dysplasia
CPAP: Continuous positive airway pressure
C/S: Cesarean section
NEC: Necrotizing enterocolitis
GA: Gestational age
HAS: Hospital acquired sepsis
HIV: Human immunodeficiency virus
IQR: Interquartile range
KMC: Kangaroo mother care
LOS: Length of hospital stay
LGA: Large for gestational age
NICU: Neonatal intensive care unit
PMA: Post menstrual age
RDS: Respiratory distress syndrome
SGA: Small for gestational age
SVD: Spontaneous vertex delivery
UoGCSH: University of Gondar Comprehensive Specialized Hospital
VLBW: Very low birth weight
